# Nanotechnology Therapy for Alzheimer′s Disease Memory Impairment Attenuation

**DOI:** 10.3390/ijms22031102

**Published:** 2021-01-22

**Authors:** Samo Ribarič

**Affiliations:** Institute of Pathophysiology, Faculty of Medicine, University of Ljubljana, 1000 Ljubljana, Slovenia; samo.ribaric@mf.uni-lj.si

**Keywords:** nanotechnology, nano particles, Alzheimer’s disease, memory impairment, animal models

## Abstract

Currently, there is no cure for Alzheimer’s disease (AD) in humans; treatment is symptomatic only. Aging of the population, together with an unhealthy diet and lifestyle, contribute to the steady, global increase of AD patients. This increase creates significant health, societal and economical challenges even for the most developed countries. AD progresses from an asymptomatic stage to a progressively worsening cognitive impairment. The AD cognitive impairment is underpinned by progressive memory impairment, an increasing inability to recall recent events, to execute recently planned actions, and to learn. These changes prevent the AD patient from leading an independent and fulfilling life. Nanotechnology (NT) enables a new, alternative pathway for development of AD treatment interventions. At present, the NT treatments for attenuation of AD memory impairment are at the animal model stage. Over the past four years, there has been a steady increase in publications of AD animal models with a wide variety of original NT treatment interventions, able to attenuate memory impairment. NT therapy development, in animal models of AD, is faced with the twin challenges of the nature of AD, a chronic impairment, unique to human, of the tau protein and A β peptides that regulate several key physiological brain processes, and the incomplete understanding of AD′s aetiology. This paper reviews the state-of-the-art in NT based treatments for AD memory impairment in animal models and discusses the future work for translation to the successful treatment of AD cognitive impairment in human.

## 1. Nanotechnology for Alzheimer’s Disease Therapy

Nanotechnology (NT) provides a new approach to develop alternative drug delivery treatments for all stages of Alzheimer’s disease (AD). NT uses particles with at least one dimension smaller than 100 nm, the nano particles (NPs) [1]. The International Organization for Standardization defines NPs as nano-objects with all three external dimensions in the nanoscale [2].

The NPs have several advantages compared to traditional drug delivery compounds. They have a very small size with a high surface-to-volume ratio that facilitates interactions with biomolecules. They can be produced to different shapes (spherical, cubic, rod-like) and sizes to modify their movement across biological barriers. NPs can be used either for disease diagnosis or for treatment. They can bind with a wide variety of desired ligands (by adsorbing, entrapping or covalent bonding) to acquire new diagnostic, therapeutic or physiological properties, including the ability to cross the blood-brain barrier (BBB) [1].

NPs, for AD treatment or diagnosis, are either natural-polymer based, synthetic-polymer based or inorganic substances. Examples of synthetic-polymer based NPs are poly (ethylenimine), poly-(alkylcyanoacrylates), poly (amidoamine) dendrimers, poly (ε-caprolactone), poly (D, L-lactic acid) (PLA), poly (lactic-co-glycolic acid) (PLGA), polyethylene glycol (PEG), and polyesters (poly (lactic acid) (PLA)). Examples of inorganic materials for therapeutic NPs are gold, silica, carbon. The desired size and shape of NPs is more difficult to achieve from polymeric than from inorganic materials. A faster degradation and elimination from the body through the kidneys, and a lower risk of toxicity make polymeric NPs more suitable for human therapeutic applications than inorganic NPs. Natural-polymer based NPs, such as amino acids (poly(lysine), poly (aspartic acid), polysaccharides (chitosan and alginate) and proteins (gelatine and albumin), have the ability to interact with specific receptors/transporters expressed by endothelial cells combined with the disadvantages of poor structure modification and tracking by imaging platforms. Inorganic NPs are most suitable for imaging applications, due to their long half-life and inherent chemical stability in the biological environment. The desired physio-chemical properties of NPs, for optimal therapeutic efficiency, are: a size between 50 and 100 nm, a spherical shape, a close to zero (low positive charge) or negative zeta potential [3].

NPs tend to adsorb proteins in body fluids and form a protein coating. For example, gold NPs adsorb more than 70 different serum proteins. This protein layer modulates the NPs′ ability to interact with the environment, changes the NPs′ physicochemical properties, aggregation rate, half-life and in case of blood borne NPs, enhances their sequestration in spleen and liver. Coating is attenuated by grafting NPs with PEG and also increases NPs′ blood half-life [3].

The BBB controls bidirectional transport of biomolecules between blood vessels and brain cells. Crossing the BBB is a significant challenge for the development of drug delivery systems to the brain. The physicochemical properties of NP are modified, by attaching different ligands, with optimal ligand density and receptor affinity, to their surface to facilitate drug delivery. Examples of ligands that facilitate BBB penetration are: (a) Ligands that interact directly with BBB receptors or transporters-poly(sorbate 80, alias, Tween 80) with adsorbed apolipoprotein E and/or A-I from the blood stream; (b) ligands with direct interaction with BBB receptors or transporters-for transferrin or insulin receptor, or glucose transporter; (c) ligands that increase the NPs′ charge and hydrophobicity-amphiphilic peptides facilitating uptake by BBB endothelial cells; and (d) ligands that improve blood circulation time-for example, PEG or PEG–PLGA [1,2,3,4,5,6,7,8,9]. Classification and properties of NPs, evaluated for AD treatment and diagnostic interventions, are presented in Table 1 [8,10,11,12,13].

**Figure 1:**
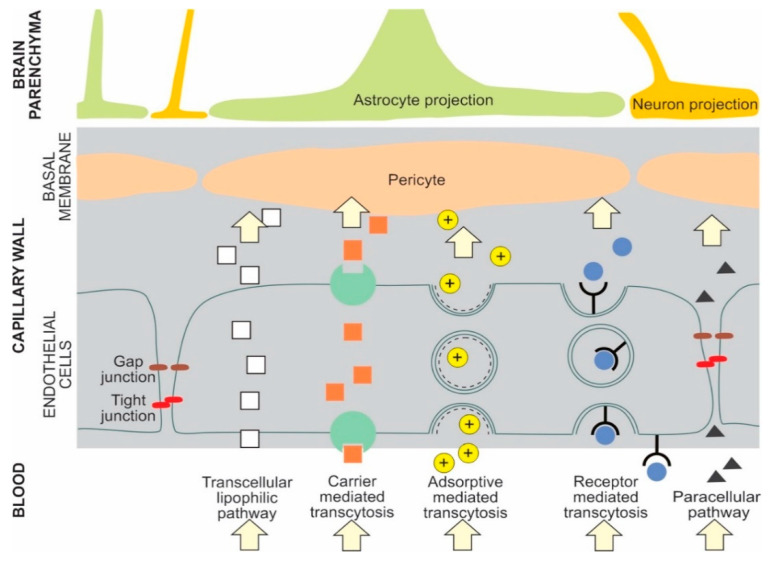
Examples of potential NP transport pathways across the blood-brain barrier.

The main transport pathways for NPs across the BBB are receptor mediated transcytosis and adsorptive mediated transcytosis [1,14] NPs can cross the BBB by several pathways. For example, liposomes can cross the BBB by either receptor mediated transcytosis or adsorptive mediated transcytosis [7]. Selected types of potential NP BBB pathways are presented in Figure 1 [1,5,6,7,15,16].

NPs are increasingly recognized as promising candidates for new AD therapies [3,4,6,17]. The amyloid cascade hypothesis still underpins the development of Aβ-related diagnosis/treatment approaches. However, research has shifted to a multifactorial aetiology approach to AD, recognising the unique temporal contributions of (a) Aβ1-42 accumulation, formation and accumulation of toxic, soluble Aβ oligomers (AβOs); (b) the binding of zinc, copper, and iron cations to Aβ1-42 peptides that accelerates formation of AβOs; (c) tau protein phosphorylation, and (d) oxidative stress and chronic neuroinflammation elicited and sustained by glial cells. The future of NP-based treatments of AD is in developing treatment interventions tailored to each of the four AD stages: (a) The asymptomatic, preclinical stage; (b) the progressively symptomatic mild cognitive impairment (MCI); (c) the mild to moderate dementia; and (d) the severe dementia [3,4,6,8].

The mechanistic approaches to development of AD therapies, with NPs carrying therapeutic agents, are: (a) the clearance of Aβ fibrils/aggregates; (b) the development of acetylcholinesterase inhibitors loaded NPs to ameliorate cholinergic system impairment; (c) the attenuation of neuroinflammation; (d) the attenuation of tau hyperphosphorylation; (e) the development of anti-Aβ peptide antibodies loaded on circulating NPs that initiate ‘the sink mechanism’, by removing the soluble Aβ peptides from the brain to the blood circulation.

## 2. Alzheimer′s Disease and Memory Impairment

### 2.1. Short-Term and Long-Term Memory

Memory formation and retrieval are essential brain functions supporting human′s daily activities. The key process enabling memory retention is the conversion of short-term memory (STM) to long-term memory (LTM). STM and LTM are formed and supported by distinct neurobiological processes. STM is underpinned by modulated activity patterns of existing brain neural networks and their post-translational modifications of proteins (e.g., protein phosphorylation). LTM is underpinned by structural and functional changes of neural networks elicited by new gene expression (e.g., an increase of the number and size of synaptic connections among specific brain neural networks) [17,18].

### 2.2. Memory Impairment

Memory impairment, including the degraded formation and recall of memories, can be present in the acute or chronic brain disorders. In human brain disorders, up to four distinct memory modalities can be affected: Episodic, semantic, working, and procedural memory —each with specific clinical signs of memory loss, affected neuroanatomical networks, and commonly associated acute or chronic disorders. Consciously recalled memories of events, objects or facts are labelled as declarative memories, and their formation is critically dependent on normal function of hippocampus and medial temporal lobes [17]. Examples of declarative memories are: Episodic, semantic, and working memories. Episodic memory impairment, the inability to recall recent personal events, is one of the most often perceived forms of memory dysfunction in human. This memory impairment is usually associated with hippocampus and medial temporal lobes dysfunction; however, other brain structures also participate in episodic memory formation: diencephalon, limbic system, posterior cingulate and precuneus region [19]. Progressive memory impairment, ultimately contributing to dementia, that is associated with pathological changes of hippocampus, medial temporal lobes, posterior cingulate or precuneus region, is a hallmark of Alzheimer’s disease [20,21,22].

### 2.3. Alzheimer′s Disease, Dementia and Memory Impairment

The most prevalent symptom of AD in humans is the cognitive impairment [23]. Also, AD is the most common cause of human dementia, a state of severe cognitive impairment affecting memory, thinking, and behaviour that prevents the patient to independently perform everyday activities. The projected number of AD patients will triple between 2013 and 2023 [24]. Two sub-groups of AD are identified. The early-onset form, clinically diagnosed before 65 years of age, a polygenic form where only 10% of the early-onset cases are attributable to the altered gene expression of either amyloid precursor protein, presenilin-1 or presenilin-2, affecting about 1% of all AD patients. The best understood early-onset forms of AD are the familial early-onset forms (efAD) with mutations in expression of amyloid precursor protein, presenilin-1 or presenilin-2. The late-onset, sporadic form (sAD), is clinically diagnosed before 65 years of age and affects 99% of all AD patients [25]. The apolipoprotein E4 (Apo-E4) gene is a known risk factor for the late-onset AD, increasing the risk by up to 10-fold [26]. Individuals with *Apo-E2* or *Apo-E3* gene have a higher synaptic plasticity and repair capacity compared to the non *Apo-E2*, non *Apo-E3* population [27]. The AD diagnosis is unequivocally confirmed only post-mortem by the brain atrophy associated with; (a) extracellular senile plaques composed of Aβ peptides in various stages of aggregation (i.e., amyloid deposits) and (b) intraneuronal neurofibrillary tangles (NFTs), composed of hyperphosphorylated tau protein. In human, these characteristic intracellular and extracellular lesions first appear in the hippocampus and entorhinal cortex (the main interface between hippocampus and neocortex). The entorhinal cortex–hippocampus system underpins episodic memories, especially the formation, consolidation, and sleep optimisation of spatial memories. Later, the AD associated lesions spread to include the temporal, parietal, and frontal association cortices [28]. The AD memory impairment is assumed to occur when the progressive reduction in brain synaptic density abolishes the cognitive reserve (CR). The CR varies in size, from person to person, and explains the variability in memory decline among AD patients with similar brain pathology. Paradoxically, a later appearance in clinical signs of dementia, due to a high CR, is followed by a faster progression of memory decline [29,30].

## 3. Potential Animal Models for Alzheimer′s Disease Memory Impairment

The fact that there is no ideal animal model for the study of AD memory impairment is reflected by the large number of animal models and consequently the lack of a standard model to compare the large body of research. Rodents, mice and rats, are the most often used animal models to study AD.

### 3.1. Transgenic Rodents

#### 3.1.1. Transgenic Mice Expressing Human *APP* (Amyloid Precursor Protein) and *PSEN1* (Presenilin 1) with efAD Mutations

Transgenic mice expressing human *APP*, with or without human *PSEN1*, have consistent brain plaque formation, gliosis, decreased levels of synaptic markers and impairment of spatial memory tasks, in the cortex and hippocampus, as is also evidenced in human AD. Mice expressing multiple FAD mutations, for example the 5XFAD model, have a more severe AD brain pathology that develops at a younger age (intraneuronal Aβ peptides accumulation at six weeks, and plaque formation at two months). Limitations of these transgenic mouse models, to follow the pathogenesis of AD in the human brain, are (a) no widespread neurodegeneration and regional brain atrophy, (b) cognitive impairment is concomitant with plaque development in mice as opposed to the human form of AD where cognitive impairment develops years later and (c) a lack of NFTs [23].

#### 3.1.2. Transgenic Mice Expressing Tau

NFTs form in brains of transgenic mice expressing human frontotemporal lobar degeneration tau mutations (e.g., *P301L* or *P301S*). Mice expressing NFTs have associated brain neurodegeneration and atrophy with movement disorders that interfere with cognitive testing. In human AD, the aforementioned tau mutations are not present, nor does tau overexpression elicit severe motor disorders [23].

#### 3.1.3. Transgenic Mice with Both Plaques and Tangles

AD transgenic animal models with brain plaques and tangles concurrently express mutated forms of *APP*, *MAPT* (encodes microtubule associated protein tau) and *PSEN1* or *PSEN2* (Presenilin 2). The most complete transgenic mouse model of AD pathology is the 3XTg mouse model that develops intraneuronal Aβ at 3–4 months, cortical and hippocampal senile plaques at 6 months and cortical and hippocampal NFTs at 12 months. Localised brain neurodegeneration, synaptic impairment and cognitive deficits are present at 6 months. The model’s drawbacks are: (a) Highly over-expressed, mutated Aβ peptides and tau that are not representative of those in sAD; and (b) the late and less widespread development of brain plaques and NFTs, not typical for the human sAD [23].

#### 3.1.4. Knock-in Mouse Models

Knock-in AD mouse models, designed by humanizing mouse Aβ peptides and knocking in specific *APP* efAD mutations, simulate better the human AD associated neuropathology with the benefit of avoiding the concurrent effects of APP over-expression. Thus, the *AP*P expression pattern follows the correct brain regions and cell types. The start of AD associated neuropathology expression is mutation specific and ranges from six to 18 months. This animal model simulates the efAD, and not the sAD [23].

#### 3.1.5. Transgenic Rat Models

The advantages of AD transgenic rat models over mouse models are: (1) Closer physiological, morphological and genetic characteristics to human; (2) larger brain volume, facilitating imaging, sample collection and electrophysiology studies; (3) formation of NFTs with endogenous rat tau (TgF344-AD rats); (4) the consistent development of AD associated cognitive impairment [23].

### 3.2. Rat Brain Injury Models of Alzheimer’s Disease

AD rat brain injury models are elicited by (a) a single, intra-cerebro-ventricular injection (i.c.v.i.) of streptozotocin (STZ) [31] or β amyloid [32], (b) an intra-peritoneal injection of scopolamine [33] or (c) a chronic administration of AlCl_3_ (aluminium chloride) [34]. I.c.v.i. of STZ promotes formation of NFTs by inhibition of phosphatase, thus indirectly increasing tau phosphorylation. However, the STZ model does not induce amyloid β plaques (Aβ-PLs) formation, only tau protein hyper-phosphorylation and brain neuroinflammation [31]. Scopolamine injection increases brain nerve cell oxidative stress, measured by increased markers of brain lipid peroxidation (e.g., malondialdehyde (MDA), and by reduced markers for antioxidant activity, e.g., catalase and GSH (glutathione) [33]. AlCl_3_ brain accumulation leads to formation of Aβ-PLs and NFTs in the rat′s cortex and hippocampus [34]; Injection of amyloid β peptide (i.e., Aβ1-42) elicited biochemical changes in brain homogenates consistent with amyloid β peptide toxic effects: increased MDA and nitrite levels, and reduced GSH levels [32].

### 3.3. Animal Models of Nanoparticle-Based Therapy That Attenuate the Effect of Alzheimer’s Disease on Memory

Animal models of NT-based therapy, that attenuate the effect of AD on memory, are summarised in Table 2, heading 4.1. and in Appendix A, Table A1. To summarise, NPs were able to attenuate the AD effect on memory in (a) mice: transgenic mice expressing human *APP* and *Tau*, transgenic mice simultaneously expressing plaques and tangles, and normal mice injected with Aβ1-42 into the brain, and in (b) rats: rats with STZ or scopolamine brain lesions, normal rats injected with amyloid β peptides (Aβ1-40, or Aβ1-42) into the brain, and rats feed with AlCl_3_.

## 4. Mouse and Rat Models Where NPs Attenuated Alzheimer’s Disease Memory Impairment

### 4.1. Animal Models of Alzheimer’s Disease Where NPs Attenuated Memory Loss

Table 2 summarises AD animal models used in the 30 reviewed papers. The preferred mouse models are transgenic mice, the preferred rat models are normal rats with AD-like brain pathology that is elicited by exposing the animals’ brains to high concentrations of either Aβ1-40, Aβ1-42, STZ, scopolamine, okadaic acid or AlCl_3_. No transgenic rats are used to evaluate the NPs effect on AD-like memory dysfunction.

### 4.2. Molecular and Cellular Effects of NPs-Based Treatments in Mouse and Rat Models Where Alzheimer′s Disease Memory Impairment Was Attenuated

**Figure 2:**
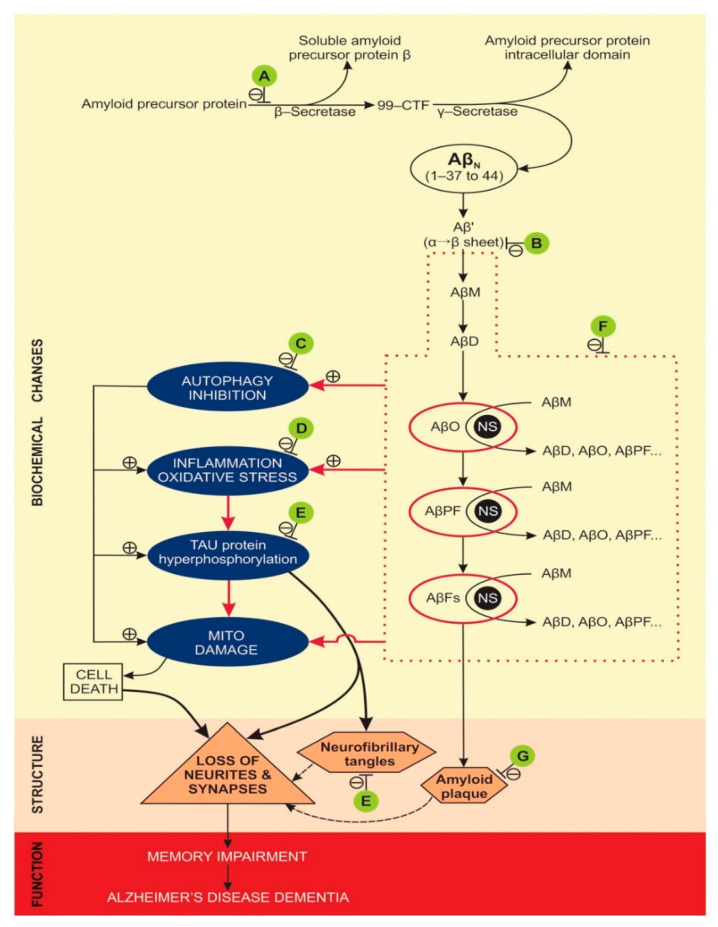
Molecular pathways of amyloid and tau pathology, where NPs treatment attenuated memory dysfunction in AD model animals. A (attenuates β-secretase): [47,57]; B (attenuates α→β sheet conversion): [42]; C (attenuates autophagy inhibition): [48]; D (attenuates inflammation): [32,33,35,36,37,39,41,43,46,49,51,53,54,55,56]; E (attenuates tau hyperphosphorylation): [13,31,43,44,49]; F (attenuates Aβ oligomerisation and fibrilization): [11,12,34,35,37,38,39,40,41,42,43,44,45,47,48,49,50,51,52,56,57]; G (attenuates amyloid plaque formation): [13,31,33,37,40,42,43,46,48,54,57]. Abbreviations: 99-CTF (99-amino acid membrane bound C-terminal fragment), Aβ’ (misfolded Aβ peptide with first α-sheet, then β-sheet structure), Aβ_N_ (native Aβ peptides with α-helix structure), AβD (amyloid β dimer), AβFs (amyloid β fibrils), AβM (amyloid β monomer), AβO (toxic, soluble amyloid β oligomer), AβPF (amyloid β protofibril), AD (Alzheimer’s disease), NFT (neurofibrillary tangles), NS (nucleation site), ⊥ attenuates/inhibits, β-S (β-secretase), γ-S (γ-secretase), ⊕ promotes/accelerates. Doted lines denote the minor contribution of neurofibrillary tangles and amyloid plaques to the loss of neurites and synapses, compared to the effects of hyperphosphorylated tau and AβOs.

Molecular and cellular effects of NPs-based treatments in animal models, where AD memory impairment was attenuated, are presented in Table 3, in Figure 2, and in Appendix A, Table A1. The most often studied molecular targets for NPs treatments were amyloid β pathology and neuroinflammation.

### 4.3. Memory Assessment Tests of NPs-Based Treatments in Mouse and Rat Models Where Alzheimer′s Disease Memory Impairment Was Attenuated

Long-term memory (LTM) assessment tests, of NPs-based treatments in AD animals, are listed in Table 4. The most often employed LTM tests were the Morris water maze test (to evaluate spatial memory), and the Novel object recognition test (to evaluate recognition memory).

## 5. Discussion

### 5.1. Development of Alzheimer’s Disease over Time

The duration of AD varies between 3–10 years, depending on several factors, including the patient’s age at the time of clinical diagnosis, lifestyle, and general health [58,59]. However, the key pathological changes in the brain, preceding the AD associated clinical signs and symptoms (e.g., memory dysfunction, depression) can develop decades earlier [60,61]. Analysis of longitudinal AD patient data suggests that the first signs of memory dysfunction can occur up to three decades before dementia. These early signs of cognitive decline are followed by abnormal changes in Aβ1-42 cerebrospinal fluid (CSF) levels and concurrent hippocampal atrophy. More than a decade after the first signs of memory dys- function, brain hypometabolism develops, accompanied by abnormal changes in total and phosphorylated tau proteins levels [62]. These conclusions are consistent with the study of predicting time to dementia in AD patients participating in the Neuroimaging Initiative that reported early changes in verbal memory, CSF Aβ1–42, and hippocampal volume [63]. Therefore, early diagnosis and treatment at the asymptomatic phase of AD seems to be vital and can be assisted by a personalised prediction of the AD progression timeline [64].

### 5.2. Molecular Mechanisms of Alzheimer’s Disease

The model of AD molecular mechanisms evolved in parallel with the in vitro and in vivo AD models and measuring methods, and with the development of diagnostic techniques for AD in human [65,66,67]. Initially, Aβ-PLs and NFTs were assumed to be the main driver of loss of neurites and synapses with subsequent memory impairment and dementia. Further discoveries of AD molecular mechanisms shifted the focus from Aβ-PLs and NFTs to AβOs as the main driver of secondary tau pathology and memory impairment in AD. Consequently, the “amyloid cascade hypothesis” was revised to the “AβOs cascade hypothesis” [65]. The current consensus is that, compared to hyperphosphorylated tau and AβOs, Aβ-PLs and NFTs are less toxic, i.e., have a smaller contribution to memory impairment [65,68]. Until recently, the consensus was that AβOs contribute the most to synaptic damage and memory deficit in AD [69]. However, the results of AD treatment, focused on attenuating the production and/or effects of AβOs were not consistent with this assumption [4,70,71,72,73,74,75]. Also, Aβ (1-40/1-42) peptides, secreted by brain cells (neurons and astrocytes) and non-neural tissues (e.g., skin, muscle, intestinal epithelium), have several physiological roles including antimicrobial, tumour suppression, regulation of BBB permeability, stimulation of brain injury recovery and synaptic function regulation [19]. Aβ peptides contribute to memory consolidation in the hippocampus, by modulating the activity of glutamatergic and cholinergic synapses [19]. Therefore, an aggressive attenuation treatment, of Aβ peptides in the brain of AD patients, could have a counterproductive effect on attenuating memory dysfunction. A recent review, summarizing experimental evidence for an alternative to the AβOs cascade hypothesis of AD, suggests tau pathology, not Aβ proteins pathology, as the principal cause for development and progression of AD. This recent version of the “tau hypothesis” postulates the existence of an amyloid precursor protein metabolic impairment (or impairments) that triggers in parallel tau pathology and Aβ pathology (accumulation of AβOs and Aβ-PLs). Tau pathology is directly responsible for neuronal and synaptic loss. Aβ pathology contributes to neuronal and synaptic loss indirectly by sustaining chronic brain inflammation that promotes tau pathology [67]. Neuroinflammation plays an important role at several stages of amyloid and tau pathology. For example, β-secretase 1 expression is stimulated by inflammatory cytokines that reduce PPAR1, an inhibitor of β-secretase 1 mRNA [57].

Therefore, current treatment developing strategies are underpinned by efforts to manage AD′s multifactorial pathogenesis, to mitigate simultaneously the parallel pathological processes of neuroinflammation (with increased release of cytotoxic hydrogen peroxide), oxidative stress, mitochondrial dysfunction, disparity of zinc and copper ions, and formation of soluble toxic AβOs and tau hyperphosphorylation [60,76,77,78,79,80,81,82,83,84].

### 5.3. Animal Models of NPs Based Therapy for Alzheimer’s Disease Associated Memory Dysfunction

Animal models used for attenuation of AD associated memory dysfunction with NPs were limited to mouse or rat models (Table 2). Mouse AD models were, either non-transgenic [35,36], where mice’s brains were injected with a high concentration of Aβ1-42, or transgenic, most frequently used were the APP/PS1 double transgenic mice [11,38,39,40,41,42,43,44,45,46,47]. Rat models of AD, to study NPs effect on memory dysfunction, were all non-transgenic (most often adult Wistar rats), where AD-like brain pathology was elicited by exposing the animals brain to high concentrations of either Aβ1-40 [44], Aβ1-42 [51,52,53], β-amyloid proteins [32], STZ [31,56,57], scopolamine [33], okadaic acid [13] or AlCl_3_ [34]. Molecular pathways of amyloid and tau pathology, where NPs’ treatment attenuated memory dysfunction in AD model animals are summarised in Figure 2.

Although different mouse or rat models were used to evaluate the effect of NPs treatment, the most often attributed causes for mitigated AD associated memory dysfunction, in the reviewed papers, were attenuated neuroinflammation, Aβ peptides aggregation, and Aβ-PL formation. Relatively fewer models evaluated the effect of tau protein attenuation on mitigating AD associated memory dysfunction, and even a smaller number of studies evaluated the combined effects of both tau and amyloid pathology attenuation on AD associated memory dysfunction (Table 5). 

The NPs ability to cross the BBB, was essential for their mitigating effect on memory impairment with four notable exceptions (Appendix A, Table A1). Biodegradable, PEGylated NPs, surface functionalized with an antibody directed against Aβ1-42, attenuated memory dysfunction via the sink-effect, by reduction of soluble Aβ1-42 and oligomer concentrations in mice brains with a concomitant increase in Aβ1-42 plasma levels [50]. CRISPR–Cas9 (RNA-guided Cas9 nuclease from the microbial clustered regularly interspaced short palindromic repeats adaptive immune system) amphiphilic NPs were injected into the hippocampal brain region of transgenic mice to achieve attenuation of memory impairment [57]. The memory impairment mitigating effect of the silica/tau-binding peptide/iron oxide and ceria/methylene blue NPs was achieved by an extra-corporeal circulation device that removed the antibody bound Aβ1-42 by external magnet at the end point of extra corporal circuit [12]. Resonantly illuminated gold NPs, generating negatively charged plasmon-activated water (PAW), endowed the orally administered PAW with anti-oxidative and anti-inflammatory effects [43].

### 5.4. Current Challenges for Nanotechnology Therapy in Animal Models of Alzheimer’s Disease

The challenges for NT therapy in animal models of AD arise mainly from the nature of AD (a chronic impairment, unique to human, of tau protein and Aβ peptides that regulate several key physiological brain processes) and from the incomplete understanding of AD’s aetiology. Current challenges for development of NT therapy in AD animal models are: (a) unresolved AD aetiology; (b) appropriate dosing and target selectivity of NP based drugs; (c) shortcomings of animal models; (d) time scale of AD therapy and (e) pharmacodynamic and pharmacodynamic drug-drug interactions between NPs based and non-NPs based drugs commonly used by AD.

#### 5.4.1. The Alzheimer’s Disease Aetiology Challenge

The precise molecular mechanism that triggers AD is not known. At present, there are two competing hypotheses, the amyloid hypothesis and the tau hypothesis. The amyloid hypothesis attributes the primary cause of AD to excessive accumulation of AβOs [65,66]. The tau hypothesis assigns the AD molecular mechanism trigger to impairments of APP metabolism with subsequent accumulation of APP C-terminal fragments and development of tau pathology (tau aggregation, formation of paired helical filaments and NFTs) [67]. Key evidence in favour of the tau hypothesis include: (a) Strongly correlated distribution of tau pathology with the extent of cognitive and clinical symptoms, (b) tau lesions precede Aβ accumulation in the brain, (c) tau spatial patterns are closely correlated to neurodegeneration patterns [67], and (d) no correlation between amyloid plaques and loss of synapses and neurons in brains of AD patients [66]. However, no successful AD drug for humans has been developed on the basis of either hypothesis [74,83,86].

#### 5.4.2. Dosing and Target Selectivity

Current NPs based therapies in AD animal models ameliorated memory impairment by attenuating either Aβ peptides aggregation and/or tau hyperphosphorylation. Since physiological levels of both tau protein and Aβ peptides are essential for normal brain synapses function in human, the maintenance of an optimal NPs based drug dosage is vital. For example, in a clinical trial of γ-secretase inhibitor Semagacestat, the symptoms of AD patients worsened [86]. The γ-secretase protease also cleaves the membrane-spanning domain of notch protein that regulates several cellular processes, including spatial learning and memory. The failure of γ-secretase inhibitors, in clinical trials with AD patients, was attributed to the collateral inhibition of notch signalling [87].

#### 5.4.3. Shortcomings of Alzheimer’s Disease Animal Models

The most widely used animal models for evaluating NP based therapy for AD are mice and rats. The fundamental disadvantage of these models is that these animals do not develop AD in the absence of artificially elicited AD-like brain lesions or genetic manipulation (e.g., overexpression of APP or PSEN1 in transgenic mice). It is not clear how well these genetically modified mice reproduce the conditions of AD in human, since no human form of AD is associated with APP or PSEN1 overexpression. Also, mice overexpressing APP or PSEN1 can have cognitive defects and die early before brain Aβ pathology is detected. Most of AD transgenic animal models simulate Aβ pathology and a very few tau pathology, i.e., NFTs. In human, AD has not been linked to any mutation in tau [23,88].

#### 5.4.4. Time Scale of NPs Based Therapies in Animal Models of Alzheimer’s Disease

In human, the duration of AD′s preclinical phase can be several decades, and the duration of disease between 3–10 years. This is considerably longer than the duration reported in the 30 reviewed NPs based therapies in animal models of AD that never exceeded 3 months, with one exception of nine months [43]. NPs′ therapeutic efficacy could degrade over time, either due to development of antibodies to NPs or due to NPs induced neurotoxicity [2]. Therefore, the efficacy of NPs therapies, to attenuate memory loss, has to be verified on a time scale of years.

#### 5.4.5. Pharmacodynamic and Pharmacodynamic Drug-Drug Interactions

AD drugs have to be administered for years, or in the case of preventive AD therapy for decades. A recent study reported an average of 6.58 medications per pharmacotherapy regimen with a potential for an average of 2.68 drug-drug interactions [89]. Also, there is no published information on the pharmacokinetic (changes altering the drug’s metabolism) and pharmacodynamic (changes altering the drug’s potency) drug-drug interactions between NPs based drugs, nor between NPs based and non-NPs based drugs commonly used by AD patients. Therefore, animal studies of these drug-drug interactions are urgently needed. In general, the pharmacokinetic and pharmacodynamic properties are determined by the NPs’ physicochemical qualities [90].

### 5.5. Specifications for the Ideal NPs Preparations to Treat the Alzheimer’s Disease Memory Dysfunction in Human

NPs based therapies, for AD memory dysfunction in human, are in the early stages of development. The relevant questions that still needs to be answered are:

(a) What are the key molecular changes that first initiate, and later sustain the progression of AD-related brain pathology in human?
(b) How can NPs regulate tau protein and Aβ1-40/-42 peptides levels without interfering with their normal functions in brain (e.g., when adjusting synaptic plasticity in response to changes in nerve activity or BBB permeability? and
(c) What will be the clinical effect of NPs treatment on memory decline in human? Will they have a cognitive reserve “enhancing effect” with a delayed start of dementia, followed by an accelerated dementia progression, or will NPs treatments also slow the rate of dementia progression?

Some design-specifications for ideal NPs-based therapies, for treatment of AD in human, are:

(a) multifunctional NPs stimulate autophagy and simultaneously attenuate several molecular pathways involved in AD pathogenesis, e.g., tau hyperphosphorylation, AβOs formation and neuroinflammation;
(b) NPs preparations are biodegradable, metabolised by the human body, or exert their effect without having to cross the BBB (e.g., via the sink-effect);
(c) long-term use of NPs does not lead to interactions with the bodies enzymes that lead to toxic modifications of NPs in the human body;
(d) NPs function as physiological buffers, preventing pathological changes in tau proteins and Aβ peptides without interfering with their physiological functions in the human body; and
(e) NPs do not interact with other NPs-treatment preparations, nor with conventional drugs used for treatment of acute or chronic diseases.

### 5.6. Suggestions for Further Work on the Animal Models of NPs Based Therapies for Attenuation of the Alzheimer’s Disease Associated Memory Dysfunction

In the past ten years, research on NPs based therapy, for attenuation of AD associated memory dysfunction, has progressed from in vitro to in vivo proof-of-principle models. Suggestions for further work on the in vivo AD animal models include:

(a) development of animal models for the late-onset, sporadic form of AD;
(b) development of animal models that evaluate dietary and lifestyle contributions to AD pathogenesis;
(c) use of a standardised memory tests battery (e.g., Morris water maze, Novel object recognition and Passive avoidance test) to better compare the effects of different NPs on memory dysfunction treatment;
(d) comparison of therapeutic efficacies for different routes of NPs′ based therapies administration (e.g., nose-to-brain *versus* BBB, *versus* sink mechanism or cleansing extra corporal systems);
(e) comparison of pharmacokinetic and pharmacodynamic profiles of different NPs based preparations.

## Figures and Tables

**Table 1:** Classification and properties of nanoparticles tested for AD treatment or diagnosis.

Nanoparticle Types	Core Structures	Surface Modifications	Cargo
3-dimensional DNA nanostructures	tetrahedral DNA nanostructures		
Carbon nanotubes	single-walled carbon nanotubes; multi-walled carbon nanotubes	anti-Tau antibody; gold & antibody-binding protein & Aβ antibody; polysorbate or phospholipid coating	acetylcholine; berberine
Carbon quantum dots	polymerised o-phenylenediamine quasispheroidal carbon based nanomaterial of quantum size;		
Dendrimers	gallic acid-triethylene glycol; cationic phosphorous dendrimers;poly-amidoamine; poly-propylene-imine	helical β-peptide foldamers; maltose; morpholine groups; tetra-maleimidopropionyl	
Gold	gold nanoparticles; gold nanorods	carboxyl groups conjugated to nanoparticles; N-terminal cysteine peptide conjugated to gold nanorods;	
Lipid nanoparticles	solid lipid nanoparticle; nanostructured lipid carrier	monoclonal antibodies to transferrin receptors on BBB; pluronic acid; polyethylene glycol and lactoferrin; polysorbate 80	BACE1 siRNA; curcumin; donepezil; galantamine; resveratrol
Liposomes	cholesterol and phosphatidyl-choline; cholesterol and 1,2-distearoyl-sn-glycero-3-phosphocholine; cholesterol and sphingomyelin	cell penetration peptides and polyethylene glycol; phosphatidic acid and Apo-E; phosphatidic acid; polyethylene glycol	curcumin; galantamine; rivastigmine
Magnetic nanoparticles	gadopentetic acid; iron oxide (Fe_3_O_4_; Fe_3_O_3_); magnetite/ceria nanoparticles; polysiloxane matrix with gadolinium chelates;	chitosan and IgG-anti-amyloid antibodies; curcumin and polyethylene glycol and polyvinylpyrrolidone; Aβ oligomers monoclonal antibodies and polyethylene glycol and nitro-L-DOPA; Aβ-antibodies and polyethylene glycol	cyclophos-phamide
Polymeric nanoparticles	Poly (lactic-co-glycolic acid); Poly (lactic acid); chitosan; amino-group-modified mesoporous silica nanoparticles; selenium-(poly-lactide-co-glycolide) nanospheres	polyethylene glycol; polysorbate 80; polyethylene glycol and Aβ-binding peptide and targeting peptide to overcome blood brain barrier; tau-binding peptide and iron oxide and ceria nanocrystals	curcumin; galantamine; rivastigmine; tacrine; methylene blue
Silver	silver nanoparticles	/	/
Sulphur	volute, tadpole or sphere-like nanoparticles	/	/

**Abbreviations:** BACE1 (β-secretase); Apo-E (apolipoprotein E); L-DOPA (L-3,4-dihydroxy-phenylalanine); siRNA (silencing RNA).

**Table 2:** Animal models of Alzheimer’s Disease in nanoparticles attenuated memory loss.

AD Animal Experimental Model	References
Mice	
Aβ1-42 induced AD model in adult nude mice	[35]
Aβ1-42 induced AD model in aged Swiss Albino mice	[36]
Transgenic mice	
5XFAD transgenic mice	[12,37,38]
APP/PS1 and C57BL/6 transgenic mice	[37]
APP/PS1 double transgenic mice	[11,38,39,40,41,42,43,44,45,46,47,48]
APP23 transgenic mice	[49]
B6; SJL-Tg (APPSWE) 2576Kha, Taconic Europe transgenic mice	[50]
Rats	
Aβ induced AD model in adult Wistar rats	[32]
AlCl_3_ induced AD model in adult Wistar rats	[34]
Aβ1-40 induced AD model in adult Sprague-Dawley rats	[44]
Aβ1-42 induced AD model in adult Wistar rats	[51,52]
Aβ1-42 induced AD model in adult Wistar rats	[53]
okadaic acid induced AD model in adult Sprague-Dawley rats	[13]
scopolamine induced AD model in adult Wistar rats	[33]
streptozotocin induced AD in adult Wistar rats	[54,55]
streptozotocin induced AD model in adult Sprague-Dawley rats	[31]

**Abbreviations**: APP/PS1 (express a Swedish (K594M/N595L) mutation of a chimeric mouse/human *APP* (*mo/huAPP695swe*), together with the human exon-9-deleted variant of *PS1* (*PS1-dE9*)); APP23 (overexpress mutant human *APP* with the Swedish mutation); B6;SJL-Tg (*APPSWE*) 2576Kha, Taconic Europe transgenic mice (express a transgene coding for 695-amino acid isoform of human Alzheimer β-amyloid (Aβ) precursor protein carrying the Swedish mutation).

**Table 3:** Summary of molecular and cellular effects of nanoparticles-based treatments that attenuated Alzheimer’s disease memory impairment in mouse or rat models.

Nanoparticle’s Number, Name & Reference	↓ Neuro-Inflammation	↓ AβP Aggregation, ↓Amyloid Plaque Formation	↓ Tau Hyper Phos-phorylation	↓ Nerve Cell Death
01-curcumin lipid-core NPs [36]	●			
02-donepezil apolipoprotein A1 reconstituted HDL NPs [35]	●	●		
03-β-sheet breaker (H102) PEG-PLA NPs [42]		●	●	
04-PEGylated NPs, with Aβ1-42 Ab [50]		●		
05-PLGA NPs with Vitamin D-binding protein [56]	●	●		●
06-CRISPR–Cas9 amphiphilic NPs [57]		●		
07-curcumin NPs [46]	❍			❍
08-D-glutathione stabilised gold NPs [45]		●		
09-dual function self-destructive nano sweeper from peptide-polymers [48]		●		
10-epigallocatechin-3-gallate/ascorbic acid PEGylated PLGA NPs [39]	●	●		
11-Magnetite/Ceria NPs [12]		❍		
12-memantine PLGA PEGylated NPs [37]	●	●		
13-NPs with BACE1 mRNA silencing gene & D-peptide [47]		●	●	
14-poly (propylene imine) dendrimers with histidine-maltose [38]		❍		❍
15-PPaRγ agonist-loaded PLGA-PEG NPs [40]		●		
16-resonantly illuminated gold NPs generating PAW [43]		●	●	
17-resveratrol selenium delivery system NPs [41]	●	●	●	●
18-sphere-like sulphur NPs [11]		❍		❍
19-zinc NPs [49]	●	●		
20-berberine polysorbate-phospholipid NPs [32]	●			
21-Diphtheria toxoid NPs [53]	●			
22-epigallocatechin-gallate NPs [34]		●	●	
23-erythropoietin solid lipid NPs [51]	●	●		
24-gold NPs [52]		●		●
25-metformin phosphatidylserine NPs [55]	●			●
26-hesperetin NPs [54]	●			
27-nicotinamide phosphatidylserine NPs [31]			●	●
28-silica/tau-binding peptide/iron oxide and ceria/MB NPs [13]	●		●	●
29-quercetin NPs [33]	●			●
30-tetrahedral DNA NPs [44]		●		●

**Abbreviations**: ● (observed in vivo effect); ❍ (observed in vitro effect); ↓ (attenuates); Aβ*p* (Aβ1-40/-42-amyloid β peptides with 40 or 42 amino acid residues); Aβ*p* (Aβ1-40/-42); BACE1 (β-secretase); CRISPR–Cas9 (RNA-guided Cas9 nuclease from the microbial clustered regularly interspaced short palindromic repeats adaptive immune system); HDL (high density lipoprotein); MB (methylene blue); NP(s) (nanoparticle(s)); PAW (plasmon-activated water); PEG (polyethylene glycol); PLA (poly(lactic acid); PLGA (poly(lactic-co-glycolic acid)); PPaRγ (peroxisome proliferator-activated receptor γ).

**Table 4:** Long term memory assessment tests of nanoparticles-based treatments in AD animal models.

Nanoparticle′s Number, Name & Reference	Morris Water Maze	Novel Object Recognition	Other
01-curcumin lipid-core NPs [36]	●		
02-donepezil apolipoprotein A1 reconstituted HDL NPs [35]	●		
03-β-sheet breaker (H102) PEG-PLA NPs [42]	●		
04-PEGylated NPs, with Aβ1-42 Ab [50]		●	
05-PLGA NPs with Vitamin D-binding protein [56]		●	
06-CRISPR–Cas9 amphiphilic NPs [57]	●		
07-curcumin NPs [46]	●	●	
08-D-glutathione stabilised gold NPs [45]	●		
09-dual function self-destructive NP from peptide-polymers [48]	●		
10-epigallocatechin-3-gallate/ascorbic acid PEGylated PLGA NPs [39]	●	●	
11-Magnetite/Ceria NPs [12]		●	
12-memantine PLGA PEGylated NPs [37]	●		
13-NPs with BACE1 mRNA silencing gene & D-peptide [47]	●		
14-poly (propylene imine) dendrimers with histidine-maltose [38]		●	
15-PPaRγ agonist-loaded PLGA-PEG NPs [40]		●	
16-resonantly illuminated gold NPs generating PAW [43]		●	
17-resveratrol selenium delivery system NPs [41]	●		
18-sphere-like sulphur NPs [11]	●		
19-zinc NPs [49]			Co
20-berberine polysorbate-phospholipid NPs [32]	●		
21-Diphtheria toxoid NPs [53]	●		
22-epigallocatechin-gallate NPs [34]	●	●	
23-erythropoietin solid lipid NPs [51]	●		
24-gold NPs [52]	●		
25-metformin phosphatidylserine NPs [55]	●		
26-hesperetin NPs [54]		●	P
27-nicotinamide phosphatidylserine NPs [31]	●		
28-silica/tau-binding peptide/iron oxide and ceria/MB NPs [13]	●		
29-quercetin NPs [33]			Co, R
30-tetrahedral DNA NPs [44]	●		

**Abbreviations**: ● (used long term memory test); BACE1 (β-secretase); Co (Conditioned avoidance response test; CRISPR–Cas9 (RNA-guided Cas9 nuclease from the microbial clustered regularly interspaced short palindromic repeats adaptive immune system); HDL (high density lipoprotein); MB (methylene blue); NP(s) (nanoparticle(s)); P (Passive avoidance response test); PAW (plasmon-activated water); PAW (plasmon-activated water); PEG (polyethylene glycol); PLA (poly(lactic acid); PLGA (poly(lactic-co-glycolic acid)); PPaRγ (peroxisome proliferator-activated receptor γ); R (Rectangular-maze test).

**Table 5:** Summary of mechanisms underlying molecular and cellular effects of NPs-based treatments.

NP’s Number, Reference	i-Core Structure, ii-Surface Modifications and iii-Cargo	Mechanisms of Nanoparticle’s Core Structure, Surface Modifications or Cargo That Attenuate Memory Loss
01-, [36]	i- lipid core (sorbitan monostearate dispersion in medium-chain triglycerides core, polymer wall from poly(epsilon-caprolactone), and polysorbate 80 micelles stabilizers) iii- curcumin.	Curcumin attenuates Aβ1-42 elicited neuroinflammation by inhibition of nuclear factor kappa B signalling pathway and reduction of proinflammatory cytokines (e.g., interleukins 1β, 6, and tumour necrosis factor α).
02-, [35]	i- apolipoprotein-A1 reconstituted high-density lipoprotein with antibody-like high Aβ1-42 binding affinity is made from phospholipid vesicles and lipid free apoA-I; iii-donepezil.	The apolipoprotein A1 reconstituted high-density lipoprotein: (a) captures Aβ1-42 and facilitates its degradation in microglial cells by the endo/lysosomal pathway and (b) releases donepezil that inhibits acetylcholinesterase activity and prolongs acetylcholine activity in brain synapses.
03-, [42]	i- poly(ethylene glycol)-poly(lactic acid); ii- TGN peptides to enable crossing the blood-brain barrier and QSH peptides that bind to Aβ1-42.	QSH peptides bind to Aβ1-42 and prevent their oligomerisation, fibrillization and plaque formation by blocking the α→β sheet conversion in Aβ1-42. Also, the nanoparticle attenuated the Aβ1-42 aggregation stimulated tau hyperphosphorylation.
04-, [50]	i- poly(ethylene glycol); ii- antibody against Aβ1-42.	The anti-Aβ1-42-functionalized NPs bound with Aβ1-42 in the blood and reduced the levels of soluble Aβ1-42 peptide and Aβ1-42 oligomers in the brain through the “sink effect”.
05-, [56]	i- D,L-lactic acid-co-glycolic acid; ii- methylpenta(oxyethyl) heptadecanoate; iii- vitamin D-binding protein.	Vitamin D-binding protein binds to Aβ peptides thus preventing (a) their oligomerisation, fibrillization and plaque formation, (b) neuro-inflammation, and (c) cell death.
06-, [57]	i- Cas9, single-guide RNAs targeting BACE1 gene, and amphiphilic R7L10 peptide formed an amphiphilic nanocomplex.	Inhibition of BACE1 gene expression attenuated Aβ1-42 secretion and accumulation of Aβ plaques.
07-, [46]	i- soluplus polymer; iii- curcumin	The improved oral bioavailability enabled sufficient brain concentrations of curcumin to attenuate neuronal cytotoxicity induced by hydrogen peroxide, copper metal ions and Aβ1-42.
08-, [45]	i-gold; ii- antioxidant tripeptides L- and D-glutathione	L- and D-glutathione, conjugated with gold NPs, attenuated aggregation of Aβ1-42 in the brain by their antioxidant effect on reactive oxygen species.
09-, [48]	i- cationic chitosan; ii- GKLVFF peptide (recognises and co-assembles with Aβ1-42 through hydrogen-bonding interactions) and Beclin-1 (stimulates autophagy) attached to polyethylene glycol.	The nanosweeper binds Aβ1-42 in the extracellular brain space, enters the cell and upregulates autophagy, enhances self and Aβ1-42 digestion, and brain parenchyma clearance of soluble and insoluble Aβ1-42 forms.
10-, [39]	i- polyethylene glycol and poly (lactic-co-glycolic acid); iii- epigallocatechin-3-gallate and ascorbic acid (to prevent epigallocate-chin-3-gallate′s oxidation).	Epigallocatechin-3-gallate attenuates accumulation of soluble and insoluble Aβ1-42 forms, inhibits expression of inflammatory interleukins, protects synapses, and increases synaptogenesis. Ascorbic acid contributes to the anti-inflammatory effects of epigallocatechin-3-gallate.
11-, [12]	i- magnetite particles core (enable magnetic isolation of captured Aβ peptides with an external magnetic field) and ceria particles shell (scavenges reactive oxygen species triggered by the experimental animal’s immune response); ii- Aβ1-42-antibodies and polyethylene glycol conjugated to the ceria shell.	The extra corporal Aβ1-42 blood cleansing system reduced Aβ peptide concentrations in the blood and brain tissue of experimental animals. The animal′s immune response, to the Aβ1-42-antibodies, was attenuated by scavenging the reactive oxygen species with the ceria particles.
12-, [37]	i- poly (lactic-co-glycolic acid); ii- polyethylene glycol surface coating; iii- memantine (binds to the N-methyl-D-aspartate receptor-operated cation channels with a low-to-moderate affinity that preserves normal receptor function in response to a physiological release of glutamate at the synapses).	Memantine binds to N-methyl-D-aspartate channels and attenuates excessive glutamate cell stimulation (by inhibiting the prolonged influx of calcium ions) and consequent neuronal death thus improving memory. The memantine loaded NPs also reduce Aβ plaques formation and neuroinflammation.
13-, [47]	i- dendrigraft poly-L-lysine; ii- polyethylene glycol and peptide RVG29 (to enable binding to *n*-acetylcholine receptors in the blood-brain barrier and brain parenchyma cells); iii- D-peptide (inhibitor of tau fibril formation) and a plasmid DNA encoding β-secretase-antisense shRNA (inhibits expression of β-secretase)	After crossing the blood-brain barrier, the NPs enter the brain cells where D-peptide and β-secretase-antisense shRNA are released from the NPs to decrease intracellular tau fibrils formation and production of Aβ soluble and insoluble forms (i.e., Aβ plaques).
14-, [38]	i- poly(propylene imine) core with a maltose-histidine shell that improves the NPs ability to cross the blood-brain barrier.	The maltose-histidine shell does not inhibit formation of Aβ1-40 fibrils; it stimulates their clumping. Formation of Aβ1-40 oligomers and neuronal death is inhibited in the presence of maltose-histidine.
15-, [40]	i- poly (lactic-co-glycolic acid) and polyethylene glycol; ii-, iii- peroxisome proliferator-activated receptor agonist, pioglitazone (to facilitate crossing of the blood-brain barrier and for anti-inflammatory effects in the brain);	Pioglitazone binds with the peroxisome proliferator-activated receptor to modulate the inflammatory response and reduce Aβ plaques formation in AD model animals.
16- [43]	i- resonantly illuminated gold NPs.	The resonantly illuminated gold NPs reduce the hydrogen bonded structure of water, creating negatively charged plasmon-activated water. This water has anti-oxidative and anti-inflammatory effects that are assumed to attenuate formation of Aβ plaques, tau hyperphosphorylation, and neuroinflammation.
17-, [41]	i- mesoporous nano-selenium; ii- borneol target, β-cyclodextrin and ferrocene nanovalves; iii-resveratrol.	The interactions with blood or intracellular esterases release borneol, enabling the passage of NPs across the blood-brain barrier. Increased concentrations of hydrogen peroxide in the brain parenchyma trigger oxidation of ferrocene. Ferrocene oxidation leads to β-cyclodextrin dissociation and release of resveratrol. Resveratrol inhibits aggregation of Aβ peptides and thus attenuates oxidative stress, and tau hyperphosphorylation in nerve cells. Resveratrol also decreases brain pro-inflammatory cytokines interleukin 6 and tumour necrosis factor α, and increases anti-inflammatory cytokines interleukins 4 and 10.
18-, [11]	i- methionine modified morphology of sphere-like sulphur NPs.	The sphere-like sulphur NPs reduced aggregation of the copper ion-Aβ peptide complexes by; (a) attenuating the interaction between Aβ monomers and copper ions; and (b) interfering with the formation of hydrogen bonds. The NPs also decreased the intracellular reactive oxygen species and attenuated the copper ion-Aβ peptide complexes mediated cell cytotoxicity.
19-, [49]	i- poly(lactic-co-glycolic acid); ii- 7 amino acid glycopeptide conjugated with poly(lactic-co-glycolic acid to enable the crossing of the blood-brain barrier; iii-zinc	The NPs reduce the size of Aβ plaques and the levels of pro-inflammatory interleukins 6 and 18, and increase the levels of anti-inflammatory interleukin 10.
20-, [32]	i- multiple-walled carbon nanotubes; ii- carboxylated polysorbate or carboxylated phospholipid coating; iii-berberine	The NPs′ attenuated memory loss effect was attributed to berberine inhibition of brain oxidative damage induced by Aβ1-42.
21-, [53]	i- chitosan capsule; iii- diphtheria toxoid (to supress the AβOs elicited unfolded protein response, a sing of endoplasmic reticulum stress). In human, the exposure to diphtheria toxoid in adulthood is associated with the significantly reduced risk of AD.	AβOs elicit the cell′s unfolded protein response due to the accumulation of undigested, abnormal protein aggregates of AβOs, mitochondrial dysfunction, oxidative stress, and disruption of calcium homeostasis. Pre-treatment with NPs containing the diphtheria toxoid is assumed to activate an alternative pathway that attenuates the toxic effects of AβOs with a concomitant down regulation of the unfolded protein response.
22-, [34]	i- polyethylene glycol and poly (D, L-lactic acid) shell; iii-epigallocatechin-gallate (has antioxidant and metal chelation properties, promotes formation of less toxic amorphous Aβ1-42 aggregates over toxic, insoluble Aβ1-42 fibrils, and inhibits formation of neurofibrillary tangles by activating the phosphoinositide 3-kinase pathway).	The epigallocatechin-gallate loaded NPs attenuated (a) formation of Aβ plaques and neurofibrillary tangles and (b) reduced oxidative stress markers (NO and reactive oxygen species) and Aβ1-42 levels in the brain.
23-, [51]	i- glycerine monostearate solid lipid NP;iii- erythropoietin (promotes neuronal survival and neurogenesis by (a) nuclear factor kappa-light-chain-enhancer activity of activated B cells stimulation, inhibition of apoptotic proteins, and (b) inhibition of lipid peroxidation, and restoration of the antioxidant enzymes cytosolic catalase and glutathione peroxidase activity).	The erythropoietin loaded NPs reduced the oxidative stress and Aβ plaques deposition in the brain due to inhibition of lipid peroxidation and restoration of the antioxidant enzymes.
24-, [52]	i- gold; ii- citrate conjugated gold NPs.	The NPs; (a) improved neuronal survival by promoting the expression of brain-derived neurotrophic factor, cyclic adenosine monophosphate response element binding protein, and stromal interaction molecules; and (b) inhibited Aβ1-42 aggregation into toxic, soluble Aβ oligomers and fibrils.
25-, [55]	i- phosphatidylserine liposome shell; iii- metformin (reduces interleukin 1β and tumour necrosis factor α elicited neuroinflammation, and oxidative stress).	Metformin loaded liposomes; (a) decreased levels of pro-inflammatory cytokines interleukin 1β, tumour necrosis factor α and transforming growth factor β; and (b) reduced neuroinflammation, and neural cell death in the brain.
26-, [54]	i- hesperetin (has a neuroprotective effect due to scavenging of hydrogen peroxide hydroxyl radicals, and due to attenuation of calcium ions level and caspase-3 activity).	Hesperetin NPs increase the brain’s antioxidant enzymes (catalase, glutathione peroxidase, glutathione reductase and superoxide dismutase), and decrease malondialdehyde (a marker for lipid peroxidation during oxidative stress).
27-, [31]	i- solid lipid from phosphatidylserine; iii- nicotinamide.	Previous research reported that nicotinamide restores cognition in AD model animals by sirtuin inhibition, and selective reduction of phosphorylated tau [85]. Nicotinamide loaded NPs are assumed to attenuate memory loss by the aforementioned mechanisms.
28-, [13]	i- amino-group-modified mesoporous silica NPs; ii- tau-binding peptide, iron oxide and ceria nanocrystals; iii- methylene blue (a tau aggregation inhibitor).	The NPs attenuated memory loss by reducing mitochondrial oxidative stress, neuroinflammation, tau hyperphosphorylation and tau aggregation, and neuronal death in the brain.
29-, [33]	i- quercetin (an antioxidant that attenuates inflammation).	The NPs’ antioxidant effect; (a) increased brain antioxidant enzymes catalase, glutathione peroxidase and glutathione reductase; (b) reduced brain malondialdehyde; and (c) reduced oxidative stress related neuronal death and neuroinflammation in the brain.
30-, [44]	i- tetrahedral DNA nanostructures.	The NPs treatment inhibited Aβ1-40 aggregation into plaques, and mitochondria triggered apoptosis in the brain.

Abbreviations: Aβ1-40/-42 (amyloid β peptides with 40 or 42 amino acid residues); BACE1 (β-secretase); Cas9 (CRISPR associated protein 9); NP (s) (nanoparticle (s)). The preferred test for memory impairment evaluation, after NPs treatment in AD model animals, is the Morris water maze test. Only a few studies used more than one test to evaluate the animal′s memory impairment, most often a combination of Morris water maze and Novel object recognition tests (Table 5).

## Abbreviations

⊕	promotes/accelerates
↓/⊥	attenuates/inhibits
γ-S	γ-secretase
β-S (*BACE1*)	β-secretase
5XFAD	5XFAD mice express human *APP* and *PSEN1* transgenes with a total of five AD-linked mutations: the Swedish (K670N/M671L), Florida (I716V), and London (V717I) mutations in *APP*, and the M146L and L286V mutations in *PSEN1*
99-CTF	99-amino acid membrane bound C-terminal fragment
Aβ-PL	amyloid beta plaque
Aβ’	misfolded Aβ peptide with first α-sheet, then β-sheet structure
Aβ1-40/-42	amyloid β peptides with 40 or 42 amino acid residues
AβD	amyloid β dimer
AβFs	amyloid β fibrils
AβM	amyloid β monomer
Aβ*n*	native Aβ peptide with α-helix structure
AβO (s)	toxic, soluble amyloid β oligomer (s)
Aβ*p*	Aβ peptides with 40 or 42 amino-acid residues
AβPF	amyloid β protofibril
AD	Alzheimer’s disease
AlCl_3_	aluminium chloride
Apo-	apolipoprotein
*APP*	encodes amyloid precursor protein
*APP23*	overexpresses mutant human *APP* with the Swedish mutation
B6; SJL-Tg(APPSWE)2576Kha	Taconic Europe transgenic mice (express a transgene coding for 695-amino acid isoform of human Alzheimer amyloid-β (Aβ) precursor protein carrying the Swedish mutation
BBB	blood-brain barrier
Co	Conditioned avoidance response test
Cas9	CRISPR associated protein 9
CR	cognitive reserve
CRISPR–Cas9	RNA-guided Cas9 nuclease from the microbial clustered regularly interspaced short palindromic repeats adaptive immune system
CSF	cerebrospinal fluid
efAD	familial early-onset forms of AD
FAD	mutation of *APP*, is associated with a loss of its synaptotrophic activity in human
GSH	glutathione
HDL	high-density lipoprotein cholesterol
i.c.v.i.	intra-cerebro-ventricular injection
i.h.i.	intra-hippocampal injection
i.n.	intra-nasal administration
i.v.i.	intra-venous injection
L-DOPA	L-3,4-dihydroxy-phenylalanine
LTM	long-term memory
M	Morris water maze test
*MAPT*	encodes microtubule associated protein tau
MCI	mild cognitive impairment
MDA	malondialdehyde, a lipid peroxidation marker
N	Novel object recognition test
NFTs	neurofibrillary tangles
NP(s)	nanoparticle(s)
NS	nucleation site
NT	nanotechnology
O	Open field test
P	Passive avoidance response test
*p*.o.	oral administration
PAW	plasmon-activated water
PEG	polyethylene glycol
PLA	poly(lactic acid)
PLGA	poly(lactic-co-glycolic acid)
PPaRγ	peroxisome proliferator-activated receptor gamma
PSEN1	presenilin 1 protein encoded by the *PSEN1* gene in humans
R	Rectangular-maze test
sAD	late-onset, sporadic form of AD
siRNA	silencing RNA
STM	short-term memory
STZ	streptozotocin
●	observed in vivo effect (in Table 3) or used memory test (in Table 5)
❍	observed in vitro effect (in Table 3)

## Appendix A

**Table A1:** Supporting data for nanoparticles elicited reduced memory loss in animal models of Alzheimer’s Disease.

Nanoparticles (NPs), Animal Model, and Treatment Regime	NPs Cross BBB	LTM Assessment	In Vivo Data Supporting NPs Elicited Reduced Memory Loss	Refs.
			Mouse animal models	
01-curcumin lipid-core NPs 10 or 1 mg/kg of NPs, p.o. for 14 days, after a single i.c.v.i. of Aβ1-42 (400 pmol/animal) to aged Swiss Albino mice.	Yes	M	Decreased levels of inflammatory cytokines in prefrontal cortex, hippocampus and serum of NPs treated mice, compared to untreated controls.	[36]
02-Donepezil apolipoprotein A1 reconstituted HDL NPs for Aβ-targeting clearance and acetylcholinesterase inhibition tested in AD model of adult nude mice with one bilateral i.h.i. of Aβ1-42, (5μL of 82μM solution), followed by an i.v.i. daily of NPs with a donepezil dose of 1mg/kg, for 4 weeks.	Yes	M	NPs treated AD model rats had less neuronal damage, attenuated acetylcholinesterase activity and less amyloid β plaques deposition in mice brains, compared to untreated controls.	[35]
			Transgenic mice animal models	
03-β-sheet breaker (H102)-loaded PEG-PLA NPs modified with TGN peptides (as the BBB ligand) and QSH peptides for enhanced Aβ1-42-binding APP/PS1 double transgenic mice treated with an i.v.i. 250 μg/kg per day, for 19-consecutive days.	Yes	M	Decreased amyloid β plaques size and number, decreased tau protein phosphorylation and reduced synaptic loss in hippocampus of NPs treated mice.	[42]
04-Biodegradable, PEGylated NPs surface functionalized with an antibody directed against Aβ1-42 B6; SJL-Tg(APPSWE)2576Kha, Taconic Europe transgenic mice treated once every other day, an i.v.i. with 100 μL (40 mg/kg of polymer) of fluorescently-labelled anti-Aβ1-42-NPs (0.8 mg/kg of antibody) for 3-weeks.	No	N	NPs promoted the sink effect: reduction of soluble Aβ1-42 and Aβ oligomer concentrations in mice brains with a concomitant increase in Aβ1-42 plasma levels.	[50]
05-Biodegradable, PLGA NPs loaded with Vitamin D-binding protein 5XFAD transgenic mice treated with an i.v.i. of NPs 2.5 mg/kg, for 4-weeks.	Yes	N	Cortical changes in mice were: reduced Aβ1-42 peptide accumulation, neuroinflammation and neuronal death.	[56]
06-CRISPR–Cas9 amphiphilic NPs 5XFAD transgenic mice treated with a single injection of 10 μL of Cas9 nanocomplex into the CA3 hippocampal region.	No	M	Hippocampus of NPs treated mice had: (a) less inflammation, reactive microglia, apoptosis; (b) a significantly decreased Bace1 expression and concomitantly reduced production of APP β-cleavage products; and (3) a reduced amyloid β plaques formation.	[57]
07-Curcumin-loaded self-nano micellizing solid dispersion system NPs APP/PS1 double transgenic mice treated p.o. in drinking water, dose of NPs equivalent to a curcumin dose of 47 mg/kg, for 3-months.	Yes	M, N	The NPs better protected cultured neuroblastoma cells against copper metal ion, H_2_O_2_, and Aβ1-42 oligomers cytotoxicity then curcumin only. NPs treated mice had memory performance comparable to younger transgenic mice when the AD-like behavioural deficit has not yet developed.	[46]
08-D-glutathione stabilised gold NPs APP/PS1 double transgenic mice treated with an i.v.i. of NPs, 25 mg/kg, every week for 4 weeks.	Yes	M	Compared to untreated mice, NPs treated animals had a reduced amyloid β plaques deposition in hippocampus.	[45]
09-dual function self-destructive NPs from peptide-polymers that capture Aβ and promote its degradation by stimulating autophagy APP/PS1 double transgenic mice treated with an i.v.i. of cyclosporine (10 μM), followed by an i.v.i. of a single dose of 200 μg·mL−1 nano sweeper for 8-consecutive days.	Yes	M	Decreased soluble and insoluble Aβ1-42 levels in mice brain homogenates due to an upregulated autophagy.	[48]
10-Epigallocatechin-3-gallate formulated as dual-drug loaded PEGylated PLGA NPs APP/PS1 double transgenic mice treated with single daily p.o. 40 mg/kg, for 3-months.	Yes	M, N	NPs treated mice had enhanced SYN staining in CA3 region of hippocampus (indicating increased synaptic expression) and untreated mice had a reduced SYN staining in the same brain region. Compared to untreated controls, NPs treated mice had reduced neuroinflammation and amyloid β plaques accumulation in hippocampus and reduced accumulation of soluble and insoluble Aβ1-42 in cortical samples. In summary, cortical changes in mice are: reduced soluble and insoluble Aβ1-42 concentration; reduced inflammation; and increased synapse density.	[39]
11-Magnetite/Ceria NPs Assemblies (MCNA-Aβ1-42 antibodies conjugated to NPs) Extra-corporeal circulation, in an anesthetised 5XFAD transgenic mouse, from femoral vein blood to jugular vein with a 150 μL min−1 flow rate, established in an. Sequestration of Aβ1-42s performed by injecting an MCNA solution (1.8 × 10−3 m [Fe]) at starting point of extracorporeal circuit and removing the antibody bound Aβ1-42 by external magnet at the end point of extra corporal circuit.	No	N	The extra corporal Aβ1-42 blood cleansing system reduced Aβ peptide concentrations in blood and brain tissue of mice.	[12]
12-Memantine loaded PLGA PEGylated NPs APP/PS1 and C57BL/6 transgenic mice treated p.o. with NPs, with a memantine therapeutic dose of 30 mg/kg on alternate days, for 2-months.	Yes	M	Reduced number of amyloid β plaques and inflammation markers in mouse brain histology samples.	[37]
13-PEGylated dendrigraft poly-L-lysine loaded NPs with BACE1 mRNA silencing gene and D-peptide to inhibit p-tau-associated fibril formation APP/PS1 double transgenic mice treated with i.v., once a week (350 μg) for 5-weeks.	Yes	M	Cortical changes in mice: reduced extracellular formation of amyloid β plaques and reduced intracellular formation of tau-fibrils.	[47]
14-Poly(propylene imine) dendrimers with histidine-maltose APP/PS1 double transgenic mice treated with NP i.n., 10 mg/kg, for 3 months.	Yes	N	Mouse brain: no significant changes in size or number of amyloid β plaques, nor in oxidative stress markers or ratio and levels of soluble Aβ42 to Aβ40. Attenuated Aβ1-42 aggregation and Aβ protein toxicity in cultured neuroblastoma cells.	[38]
15-PPaRγ agonist-loaded PLGA-PEG NPs APP/PS1 double transgenic mice treated with 10 mg/kg administered p.o., once a day, 5 days per week, for 4-weeks.	Yes	N	amyloid β plaques deposition was reduced in mouse cortex.	[40]
16-Resonantly illuminated gold NPs generating negatively charged plasmon-activated water (PAW) APP/PS1 double transgenic mice treated p.o. ad libitum, for 9 months with PAW generated with NPs that reduce the hydrogen- bonded structure of water, giving PAW anti-oxidative and anti-inflammatory effects.	No	N	Compared to untreated transgenic mice, PAW treated mice had a reduced amyloid β plaques and *p*-tau burden in the hippocampus. PAW treatment reduced the levels of pro-inflammatory cytokines IL-1β and IL-6 in animals’ brains, however, the reduction was not statistically significant.	[43]
17-Resveratrol loaded mesoporous nano-selenium release delivery system based on borneol target, β-cyclodextrin nano valves APP/PS1 double transgenic mice treated with an i.v.i. of 1 mg/kg per day, repeated for 14-days.	Yes	M	Brain histology of NPs treated mice showed reduced amyloid β plaques formation, tau hyperphosphorylation and loss of neurons (i.e., an increased number of Nissl bodies).	[41]
18-Sphere-like sulphur nanoparticles RVG@Met@SNPs APP/PS1 double transgenic mice treated with an i.v.i. of 5.0 mg/kg, 2-times per week (Monday and Thursday), 8 injections in 4-weeks.	Yes	M	In a cell model, NPs significantly reduced Aβ1-42 self-aggregation and, by absorption of Cu 2+, aggregation of Aβ petide−Cu2+ complex. The brain location of NPs in vivo was confirmed with mouse real-time imaging combined with X-ray location.	[11]
19-Zinc loaded NPs APP23 transgenic mice received 2 daily i.p. of NPs (total daily amount of 392 μg Zn) for 14-consecutive days.	Yes	Co	Brain histology of NPs treated mice showed amyloid β plaques with a significantly decreased size. Brain expression levels of proinflammatory interleukins were significantly decreased and anti-inflammatory interleukin expression levels were comparable to normal mice controls. However, no significant change in brain Aβ fibrillary or oligomer levels in NPs treated mice.	[49]
			Rat animal models	
20-Berberine-loaded multiwalled carbon nanotubes with polysorbate and phospholipid coating AD model induced in adult Wistar rats by a single β amyloid i.c.v.i. followed by NPs treatment with an i.v.i. of NPs equivalent to 10 mg/kg of berberine.	Yes	M	Greater changes, consistent with β amyloid peptides toxic effect, in brain homogenates of NPs untreated animals (more increased malondialdehyde and nitrite levels, and more reduced glutathione levels) compared to NPs treated rats.	[32]
21-Diphtheria toxoid NPs AD model in adult Wistar rats with a single i.c.v.i. of Aβ oligomers (10 μL of 1 μg/μL), 14 days after a single i.n. NPs treatment of 15 Lf diphtheria vaccine in 40μL volume.	Yes	M	Compared to untreated AD model controls, NPs inhibited XBP-1 mRNA gene splicing (an early marker for ER stress, elicited by the presence of Aβ oligomers that elicit neuroinflammation, mitochondrial dysfunction, oxidative stress, and apoptosis.	[53]
22-pigallocatechin-gallate loaded NPs, AD model in adult Wistar rats after chronic administration of AlCl_3_ (100 mg/kg p.o. for 60 days), followed by NPs treatment 10 mg/kg per day, p.o., for 30 days.	Yes	M, N	The quantity of brain markers for AlCl_3_ brain lesions, amyloid β plaques and neurofibrillary tangles, was significantly reduced in NPs treated rats.	[34]
23-Erythropoietin solid lipid NPs AD model in adult Wistar rats with one bilateral i.h.i. of Aβ1-42 (concentration 0.5 μg/μL, 2 μL of suspension per site) on day 1, followed by NPs treatment with a single i.p. dose of 1250 IU/kg or 2500 IU/kg on days 2, 4, 6, 8, 10 and 12.	Yes	M	NPs treated animals, at both daily doses, reduced (a) oxidative stress, (b) ADP/ATP ration, and amyloid β plaques deposition in rat′s hippocampus, compared to untreated controls.	[51]
24-Gold NPs AD model in adult Wistar rats with one bilateral i.h.i. of Aβ1-42 (1 μL of 1 μg/μL to each side) on day one, followed by NPs treatment, i.p. 200 μg/mL/kg on days 4, 8, 12 and 16.	Yes	M	Hippocampal changes in NPs treated AD model rats are (a) an improved nerve survival, measured by increased BDNF, CREB, STIM1 and 2 expression; and (b) a reduced size and number of amyloid β plaques.	[52]
25-Metformin phosphatidylserine NPs NPs treatment of adult Wistar rats with 50 mg/kg, i.p., for 16 days, and a single i.c.v.i. of STZ (3 mg/kg) on day 2.	Yes	M	NPs attenuated the STZ elicited (a) the increase in cytokine levels IL1-β, TNF-α, and TGF-β and (b) nerve cell death and degeneration in NPs, compared to control.	[55]
26-Nano-hesperetin STZ induced model of AD in adult Wistar rats with a single i.c.v.i. of STZ (3 mg/kg) and NP treatment with 20 mg/kg, p.o., daily, for 3-weeks.	Yes	N, P	Rat hippocampal area: increased activity of antioxidant enzymes superoxide dismutase, glutathione peroxidase, glutathione reductase and catalase.	[54]
27-Nicotinamide loaded solid lipid NPs functionalized with phosphatidylserine STZ induced model of AD in adult Sprague-Dawley rats with a bilateral i.c.v.i. of 3 mg/kg repeated after 48 h and followed by NP treatment. i.p. 200 mg/kg per day, every other day, 4 injections in total.	Yes	M	Brain histology of NPs treated rats showed a reduced tau hyperphosphorylation, and a reduced number of apoptotic of neurons.	[31]
28-Silica/iron oxide and ceria/tau binding peptide NPs, loaded with a tau aggregation inhibitor methylene blue Okadaic acid induced AD model in adult Sprague-Dawley rats with a single, unilateral i.h.i. (300 ng in 1.5 μL of saline), followed 5 days later by NPs treatment with a single unilateral, i.h.i. of 10 μL silica/iron oxide and ceria/tau binding peptide NPs	Yes	M	NPs treatment attenuated brain neuroinflammation and tau hyperphosphorylation, compared to untreated control.	[13]
29-Quercetin NPs AD model in adult Wistar rats with a single injection of scopolamine followed by NPs treatment 30 mg/kg p.o., for 8-consecutive days.	Yes	Co, R	NPs treatment of rats: (a) attenuated the rise in scopolamine-associated biochemical (malondialdehyde lipid peroxidation and AChE levels) and morphological (gliosis) brain damage markers and (b) enabled near to normal levels of catalase and glutathione.	[33]
30-Tetrahedral DNA NPs AD model in adult Sprague-Dawley rats with one bilateral i.h.i. of Aβ1-40 (10 μL of 1 μg/μL), followed by NPs treatment i.v. 100 μL daily for 21 days.	Yes	M	Compared to untreated AD model controls, the hippocampus of NPs treated rats had (a) a higher number of neurons, (b) a reduced amyloid β plaques deposition and (c) reduced expression levels of pro-apoptotic signalling molecules caspase-3 and Bax and a normalised expression level of the anti-apoptotic Bcl-2.	[44]

Abbreviations: Co (Conditioned avoidance response test); i.c.v.i. (intra-cerebro-ventricular injection); i.h.i. (intra-hippocampal injection); i.n. (intra-nasal administration); i.v.i. (intra-venous injection); LTM (long-term memory); M (Morris water maze test); N (Novel object recognition test); p.o. (oral administration); P (Passive avoidance response test); PAW (plasmon-activated water); Refs. (references); R (Rectangular-maze test); STZ (streptozocin).

## References

[1] Athira S., Nadukkandy P., Mohanan P. (2018). Interaction of nanoparticles with central nervous system and its consequences. Am. J. Res. Med. Sci..

[2] Teleanu D.M., Chircov C., Grumezescu A.M., Volceanov A., Teleanu R.I. (2018). Impact of nanoparticles on brain health: An up to date overview. J. Clin. Med..

[3] Saraiva C., Praca C., Ferreira R., Santos T., Ferreira L., Bernardino L. (2016). Nanoparticle-mediated brain drug delivery: Overcoming blood-brain barrier to treat neurodegenerative diseases. J. Control. Release.

[4] Hajipour M.J., Santoso M.R., Rezaee F., Aghaverdi H., Mahmoudi M., Perry G. (2017). Advances in alzheimer’s diagnosis and therapy: The implications of nanotechnology. Trends Biotechnol..

[5] Pires P.C., Santos A.O. (2018). Nanosystems in nose-to-brain drug delivery: A review of non-clinical brain targeting studies. J. Control. Release.

[6] Gupta J., Fatima M.T., Islam Z., Khan R.H., Uversky V.N., Salahuddin P. (2019). Nanoparticle formulations in the diagnosis and therapy of alzheimer’s disease. Int. J. Biol. Macromol..

[7] Fonseca-Santos B., Gremiao M.P., Chorilli M. (2015). Nanotechnology-based drug delivery systems for the treatment of alzheimer’s disease. Int. J. Nanomed..

[8] de la Torre C., Cena V. (2018). The delivery challenge in neurodegenerative disorders: The nanoparticles role in alzheimer’s disease therapeutics and diagnostics. Pharmaceutics.

[9] Wong K.H., Riaz M.K., Xie Y.N., Zhang X., Liu Q., Chen H.J., Bian Z.X., Chen X.Y., Lu A.P., Yang Z.J. (2019). Review of current strategies for delivering alzheimer’s disease drugs across the blood-brain barrier. Int. J. Mol. Sci..

[10] Greish K., Alqahtani A.A., Alotaibi A.F., Abdulla A.M., Bukelly A.T., Alsobyani F.M., Alharbi G.H., Alkiyumi I.S., Aldawish M.M., Alshahrani T.F. (2019). The effect of silver nanoparticles on learning, memory and social interaction in balb/c mice. Int. J. Env. Res. Public Health.

[11] Sun J., Xie W., Zhu X., Xu M., Liu J. (2018). Sulfur nanoparticles with novel morphologies coupled with brain-targeting peptides rvg as a new type of inhibitor against metal-induced abeta aggregation. ACS Chem. Neurosci..

[12] Kim D., Kwon H.J., Hyeon T. (2019). Magnetite/ceria nanoparticle assemblies for extracorporeal cleansing of amyloid-beta in alzheimer’s disease. Adv. Mater..

[13] Chen Q., Du Y., Zhang K., Liang Z., Li J., Yu H., Ren R., Feng J., Jin Z., Li F. (2018). Tau-targeted multifunctional nanocomposite for combinational therapy of alzheimer’s disease. ACS Nano.

[14] Chenthamara D., Subramaniam S., Ramakrishnan S.G., Krishnaswamy S., Essa M.M., Lin F.H., Qoronfleh M.W. (2019). Therapeutic efficacy of nanoparticles and routes of administration. Biomater. Res..

[15] Pulgar V.M. (2018). Transcytosis to cross the blood brain barrier, new advancements and challenges. Front. Neurosci..

[16] Moura R.P., Martins C., Pinto S., Sousa F., Sarmento B. (2019). Blood-brain barrier receptors and transporters: An insight on their function and how to exploit them through nanotechnology. Expert. Opin. Drug. Deliv..

[17] Bisaz R., Travaglia A., Alberini C.M. (2014). The neurobiological bases of memory formation: From physiological conditions to psychopathology. Psychopathology.

[18] Mayford M., Siegelbaum S.A., Kandel E.R. (2012). Synapses and memory storage. Cold. Spring. Harb. Perspect. Biol..

[19] Brothers H.M., Gosztyla M.L., Robinson S.R. (2018). The physiological roles of amyloid-beta peptide hint at new ways to treat alzheimer’s disease. Front. Aging Neurosci..

[20] Matthews B.R. (2015). Memory dysfunction. Continuum.

[21] Sperling R.A., Dickerson B.C., Pihlajamaki M., Vannini P., LaViolette P.S., Vitolo O.V., Hedden T., Becker J.A., Rentz D.M., Selkoe D.J. (2010). Functional alterations in memory networks in early alzheimer’s disease. Neuromol. Med..

[22] de Ipolyi A.R., Rankin K.P., Mucke L., Miller B.L., Gorno-Tempini M.L. (2007). Spatial cognition and the human navigation network in ad and mci. Neurology.

[23] Drummond E., Wisniewski T. (2017). Alzheimer’s disease: Experimental models and reality. Acta Neuropathol..

[24] Wimo A., Jonsson L., Winblad B. (2006). An estimate of the worldwide prevalence and direct costs of dementia in 2003. Dement. Geriatr. Cogn. Disord..

[25] Wingo T.S., Cutler D.J., Wingo A.P., Le N.A., Rabinovici G.D., Miller B.L., Lah J.J., Levey A.I. (2019). Association of early-onset alzheimer disease with elevated low-density lipoprotein cholesterol levels and rare genetic coding variants of apob. JAMA Neurol..

[26] Mayeux R. (1998). Gene-environment interaction in late-onset alzheimer disease: The role of apolipoprotein-epsilon4. Alzheimer Dis. Assoc. Disord..

[27] Bufill E., Carbonell E. (2006). Apolipoprotein e polymorphism and neuronal plasticity. Am. J. Hum. Biol..

[28] Holger J. (2013). Memory loss in alzheimer’s disease. Clin. Res..

[29] Terry R.D., Katzman R. (2001). Life span and synapses: Will there be a primary senile dementia?. Neurobiol. Aging.

[30] van Loenhoud A.C., Wink A.M., Groot C., Verfaillie S.C.J., Twisk J., Barkhof F., van Berckel B., Scheltens P., van der Flier W.M., Ossenkoppele R. (2017). A neuroimaging approach to capture cognitive reserve: Application to alzheimer’s disease. Hum. Brain Mapp..

[31] Vakilinezhad M.A., Amini A., Akbari Javar H., Baha’addini Beigi Zarandi B.F., Montaseri H., Dinarvand R. (2018). Nicotinamide loaded functionalized solid lipid nanoparticles improves cognition in alzheimer’s disease animal model by reducing tau hyperphosphorylation. DARU.

[32] Lohan S., Raza K., Mehta S.K., Bhatti G.K., Saini S., Singh B. (2017). Anti-alzheimer’s potential of berberine using surface decorated multi-walled carbon nanotubes: A preclinical evidence. Int. J. Pharm..

[33] Palle S., Neerati P. (2017). Quercetin nanoparticles attenuates scopolamine induced spatial memory deficits and pathological damages in rats. Bull. Fac. Pharm. Cairo Univ. Cairo Univ..

[34] Singh N.A., Bhardwaj V., Ravi C., Ramesh N., Mandal A.K.A., Khan Z.A. (2018). Egcg nanoparticles attenuate aluminum chloride induced neurobehavioral deficits, beta amyloid and tau pathology in a rat model of alzheimer’s disease. Front. Aging Neurosci..

[35] Zhang H., Zhao Y., Yu M., Zhao Z., Liu P., Cheng H., Ji Y., Jin Y., Sun B., Zhou J. (2019). Reassembly of native components with donepezil to execute dual-missions in alzheimer’s disease therapy. J. Control Release.

[36] Giacomeli R., Izoton J.C., Dos Santos R.B., Boeira S.P., Jesse C.R., Haas S.E. (2019). Neuroprotective effects of curcumin lipid-core nanocapsules in a model alzheimer’s disease induced by beta-amyloid 1-42 peptide in aged female mice. Brain Res..

[37] Sanchez-Lopez E., Ettcheto M., Egea M.A., Espina M., Cano A., Calpena A.C., Camins A., Carmona N., Silva A.M., Souto E.B. (2018). Memantine loaded plga pegylated nanoparticles for alzheimer’s disease: In vitro and in vivo characterization. J. Nanobiotechnol..

[38] Aso E., Martinsson I., Appelhans D., Effenberg C., Benseny-Cases N., Cladera J., Gouras G., Ferrer I., Klementieva O. (2019). Poly(propylene imine) dendrimers with histidine-maltose shell as novel type of nanoparticles for synapse and memory protection. Nanomedicine.

[39] Cano A., Ettcheto M., Chang J.H., Barroso E., Espina M., Kuhne B.A., Barenys M., Auladell C., Folch J., Souto E.B. (2019). Dual-drug loaded nanoparticles of epigallocatechin-3-gallate (egcg)/ascorbic acid enhance therapeutic efficacy of egcg in a appswe/ps1de9 alzheimer’s disease mice model. J. Control Release.

[40] Silva-Abreu M., Calpena A.C., Andres-Benito P., Aso E., Romero I.A., Roig-Carles D., Gromnicova R., Espina M., Ferrer I., Garcia M.L. (2018). Ppargamma agonist-loaded plga-peg nanocarriers as a potential treatment for alzheimer’s disease: In vitro and in vivo studies. Int. J. Nanomed..

[41] Sun J., Wei C., Liu Y., Xie W., Xu M., Zhou H., Liu J. (2019). Progressive release of mesoporous nano-selenium delivery system for the multi-channel synergistic treatment of alzheimer’s disease. Biomaterials.

[42] Zheng X., Zhang C., Guo Q., Wan X., Shao X., Liu Q., Zhang Q. (2017). Dual-functional nanoparticles for precise drug delivery to alzheimer’s disease lesions: Targeting mechanisms, pharmacodynamics and safety. Int. J. Pharm..

[43] Cheng C.H., Lin K.J., Hong C.T., Wu D., Chang H.M., Liu C.H., Hsiao I.T., Yang C.P., Liu Y.C., Hu C.J. (2019). Plasmon-activated water reduces amyloid burden and improves memory in animals with alzheimer’s disease. Sci. Rep..

[44] Shao X., Cui W., Xie X., Ma W., Zhan Y., Lin Y. (2020). Treatment of alzheimer’s disease with framework nucleic acids. Cell Prolif..

[45] Hou K., Zhao J., Wang H., Li B., Li K., Shi X., Wan K., Ai J., Lv J., Wang D. (2020). Chiral gold nanoparticles enantioselectively rescue memory deficits in a mouse model of alzheimer’s disease. Nat. Commun..

[46] Parikh A., Kathawala K., Li J., Chen C., Shan Z., Cao X., Zhou X.F., Garg S. (2018). Curcumin-loaded self-nanomicellizing solid dispersion system: Part ii: In vivo safety and efficacy assessment against behavior deficit in alzheimer disease. Drug Deliv. Transl. Res..

[47] Liu Y., Huang R.Q., Han L., Ke W.L., Shao K., Ye L.Y., Lou J.N., Jiang C. (2009). Brain-targeting gene delivery and cellular internalization mechanisms for modified rabies virus glycoprotein rvg29 nanoparticles. Biomaterials.

[48] Luo Q., Lin Y.X., Yang P.P., Wang Y., Qi G.B., Qiao Z.Y., Li B.N., Zhang K., Zhang J.P., Wang L. (2018). A self-destructive nanosweeper that captures and clears amyloid beta-peptides. Nat. Commun..

[49] Vilella A., Belletti D., Sauer A.K., Hagmeyer S., Sarowar T., Masoni M., Stasiak N., Mulvihill J.J.E., Ruozi B., Forni F. (2018). Reduced plaque size and inflammation in the app23 mouse model for alzheimer’s disease after chronic application of polymeric nanoparticles for cns targeted zinc delivery. J. Trace. Elem. Med. Biol..

[50] Carradori D., Balducci C., Re F., Brambilla D., Le Droumaguet B., Flores O., Gaudin A., Mura S., Forloni G., Ordonez-Gutierrez L. (2018). Antibody-functionalized polymer nanoparticle leading to memory recovery in alzheimer’s disease-like transgenic mouse model. Nanomedicine.

[51] Dara T., Vatanara A., Sharifzadeh M., Khani S., Vakilinezhad M.A., Vakhshiteh F., Nabi Meybodi M., Sadegh Malvajerd S., Hassani S., Mosaddegh M.H. (2019). Improvement of memory deficits in the rat model of alzheimer’s disease by erythropoietin-loaded solid lipid nanoparticles. Neurobiol. Learn. Mem..

[52] Sanati M., Khodagholi F., Aminyavari S., Ghasemi F., Gholami M., Kebriaeezadeh A., Sabzevari O., Hajipour M.J., Imani M., Mahmoudi M. (2019). Impact of gold nanoparticles on amyloid beta-induced alzheimer’s disease in a rat animal model: Involvement of stim proteins. ACS Chem. Neurosci..

[53] Heydari S., Hedayati Ch M., Saadat F., Abedinzade M., Nikokar I., Aboutaleb E., Khafri A., Mokarram A.R. (2020). Diphtheria toxoid nanoparticles improve learning and memory impairment in animal model of alzheimer’s disease. Pharmacol. Rep..

[54] Kheradmand E., Hajizadeh Moghaddam A., Zare M. (2018). Neuroprotective effect of hesperetin and nano-hesperetin on recognition memory impairment and the elevated oxygen stress in rat model of alzheimer’s disease. Biomed. Pharmacother..

[55] Saffari P.M., Alijanpour S., Takzaree N., Sahebgharani M., Etemad-Moghadam S., Noorbakhsh F., Partoazar A. (2020). Metformin loaded phosphatidylserine nanoliposomes improve memory deficit and reduce neuroinflammation in streptozotocin-induced alzheimer’s disease model. Life Sci..

[56] Jeon S.G., Cha M.Y., Kim J.I., Hwang T.W., Kim K.A., Kim T.H., Song K.C., Kim J.J., Moon M. (2019). Vitamin d-binding protein-loaded plga nanoparticles suppress alzheimer’s disease-related pathology in 5xfad mice. Nanomedicine.

[57] Park H., Oh J., Shim G., Cho B., Chang Y., Kim S., Baek S., Kim H., Shin J., Choi H. (2019). In vivo neuronal gene editing via crispr-cas9 amphiphilic nanocomplexes alleviates deficits in mouse models of alzheimer’s disease. Nat. Neurosci..

[58] Norton S., Matthews F.E., Barnes D.E., Yaffe K., Brayne C. (2014). Potential for primary prevention of alzheimer’s disease: An analysis of population-based data. Lancet Neurol..

[59] Zanetti O., Solerte S.B., Cantoni F. (2009). Life expectancy in alzheimer’s disease (ad). Arch. Gerontol. Geriatr..

[60] Bateman R.J., Xiong C., Benzinger T.L., Fagan A.M., Goate A., Fox N.C., Marcus D.S., Cairns N.J., Xie X., Blazey T.M. (2012). Clinical and biomarker changes in dominantly inherited alzheimer’s disease. N. Engl. J. Med..

[61] Lesne S.E., Sherman M.A., Grant M., Kuskowski M., Schneider J.A., Bennett D.A., Ashe K.H. (2013). Brain amyloid-beta oligomers in ageing and alzheimer’s disease. Brain.

[62] Hadjichrysanthou C., Evans S., Bajaj S., Siakallis L.C., McRae-McKee K., de Wolf F., Anderson R.M., Alzheimer’s Disease Neuroimaging Initiative (2020). The dynamics of biomarkers across the clinical spectrum of alzheimer’s disease. Alzheimer’s Res. Ther..

[63] Bilgel M., Jedynak B.M. (2019). Predicting time to dementia using a quantitative template of disease progression. Alzheimer’s Dement..

[64] Venkatraghavan V., Bron E.E., Niessen W.J., Klein S., Alzheimer’s Disease Neuroimaging Initiative (2019). Disease progression timeline estimation for alzheimer’s disease using discriminative event based modeling. Neuroimage.

[65] Huang Y.R., Liu R.T. (2020). The toxicity and polymorphism of beta-amyloid oligomers. Int. J. Mol. Sci..

[66] Chen G.F., Xu T.H., Yan Y., Zhou Y.R., Jiang Y., Melcher K., Xu H.E. (2017). Amyloid beta: Structure, biology and structure-based therapeutic development. Acta Pharmacol. Sin..

[67] Kametani F., Hasegawa M. (2018). Reconsideration of amyloid hypothesis and tau hypothesis in alzheimer’s disease. Front. Neurosci-Switz..

[68] Spires-Jones T.L., Kopeikina K.J., Koffie R.M., de Calignon A., Hyman B.T. (2011). Are tangles as toxic as they look?. J. Mol. Neurosci..

[69] Haass C., Selkoe D.J. (2007). Soluble protein oligomers in neurodegeneration: Lessons from the alzheimer’s amyloid beta-peptide. Nat. Rev. Mol. Cell Biol..

[70] Abbott A., Dolgin E. (2016). Failed alzheimer’s trial does not kill leading theory of disease. Nature.

[71] Imbimbo B.P. (2009). Why did tarenflurbil fail in alzheimer’s disease?. J. Alzheimer’s Dis..

[72] Reardon S. (2015). Alzheimer antibody drugs show questionable potential. Nat. Rev. Drug Discov..

[73] Cummings J.L., Morstorf T., Zhong K. (2014). Alzheimer’s disease drug-development pipeline: Few candidates, frequent failures. Alzheimer’s Res. Ther..

[74] Doody R.S., Thomas R.G., Farlow M., Iwatsubo T., Vellas B., Joffe S., Kieburtz K., Raman R., Sun X., Aisen P.S. (2014). Phase 3 trials of solanezumab for mild-to-moderate alzheimer’s disease. N. Engl. J. Med..

[75] Salloway S., Sperling R., Fox N.C., Blennow K., Klunk W., Raskind M., Sabbagh M., Honig L.S., Porsteinsson A.P., Ferris S. (2014). Two phase 3 trials of bapineuzumab in mild-to-moderate alzheimer’s disease. N. Engl. J. Med..

[76] Reiman E.M., Quiroz Y.T., Fleisher A.S., Chen K., Velez-Pardo C., Jimenez-Del-Rio M., Fagan A.M., Shah A.R., Alvarez S., Arbelaez A. (2012). Brain imaging and fluid biomarker analysis in young adults at genetic risk for autosomal dominant alzheimer’s disease in the presenilin 1 e280a kindred: A case-control study. Lancet Neurol..

[77] Corbett A., Pickett J., Burns A., Corcoran J., Dunnett S.B., Edison P., Hagan J.J., Holmes C., Jones E., Katona C. (2012). Drug repositioning for alzheimer’s disease. Nat. Rev. Drug Discov..

[78] Mapstone M., Cheema A.K., Fiandaca M.S., Zhong X., Mhyre T.R., MacArthur L.H., Hall W.J., Fisher S.G., Peterson D.R., Haley J.M. (2014). Plasma phospholipids identify antecedent memory impairment in older adults. Nat. Med..

[79] Hye A., Lynham S., Thambisetty M., Causevic M., Campbell J., Byers H.L., Hooper C., Rijsdijk F., Tabrizi S.J., Banner S. (2006). Proteome-based plasma biomarkers for alzheimer’s disease. Brain.

[80] ManafiRad A., Farzadfar F., Habibi L., Azhdarzadeh M., Aghaverdi H., Tehrani K.H., Lotfi M., Kehoe P.G., Sheidaei A., Ghasemian A. (2014). Is amyloid-beta an innocent bystander and marker in alzheimer’s disease? Is the liability of multivalent cation homeostasis and its influence on amyloid-beta function the real mechanism?. J. Alzheimer’s Dis..

[81] Pratico D. (2008). Oxidative stress hypothesis in alzheimer’s disease: A reappraisal. Trends Pharmacol. Sci..

[82] Bouayed J., Rammal H., Soulimani R. (2009). Oxidative stress and anxiety: Relationship and cellular pathways. Oxid. Med. Cell. Longev..

[83] Drummond E., Goni F., Liu S., Prelli F., Scholtzova H., Wisniewski T. (2018). Potential novel approaches to understand the pathogenesis and treat alzheimer’s disease. J. Alzheimer’s. Dis..

[84] Milisav I., Suput D., Ribaric S. (2015). Unfolded protein response and macroautophagy in alzheimer’s, parkinson’s and prion diseases. Molecules.

[85] Green K.N., Steffan J.S., Martinez-Coria H., Sun X., Schreiber S.S., Thompson L.M., LaFerla F.M. (2008). Nicotinamide restores cognition in alzheimer’s disease transgenic mice via a mechanism involving sirtuin inhibition and selective reduction of thr231-phosphotau. J. Neurosci..

[86] Doody R.S., Raman R., Farlow M., Iwatsubo T., Vellas B., Joffe S., Kieburtz K., He F., Sun X., Thomas R.G. (2013). A phase 3 trial of semagacestat for treatment of alzheimer’s disease. N. Engl. J. Med..

[87] De Strooper B. (2014). Lessons from a failed gamma-secretase alzheimer trial. Cell.

[88] King A. (2018). The search for better animal models of alzheimer’s disease. Nature.

[89] Niu J., Straubinger R.M., Mager D.E. (2019). Pharmacodynamic drug-drug interactions. Clin. Pharmacol. Ther..

[90] Kang H., Mintri S., Menon A.V., Lee H.Y., Choi H.S., Kim J. (2015). Pharmacokinetics, pharmacodynamics and toxicology of theranostic nanoparticles. Nanoscale.

